# Interference figures of polarimetric interferometry analysis of the human corneal stroma

**DOI:** 10.1371/journal.pone.0178397

**Published:** 2017-06-01

**Authors:** Rodolfo Mastropasqua, Mario Nubile, Niccolò Salgari, Manuela Lanzini, Roberta Calienno, Peter A. Mattei, Alessandra Sborgia, Luca Agnifili

**Affiliations:** 1 Ophthalmology Unit, Department of Neurological, Neuropsychological, Morphological and Movement Sciences, University of Verona, Verona, Italy; 2 National Centre of High Technology (CNAT) in Ophthalmology of University “G. d’Annunzio”, Chieti-Pescara, Italy; 3 Ophthalmology Department, University of Bari, Bari, Italy; Instituto Butantan, BRAZIL

## Abstract

A rotating polarimetric 90°-cross linear-filter interferometry system was used to detect the morphological characteristics and features of interference patterns produced in in-vivo corneal stroma in healthy human corneas of 23 subjects. The characteristic corneal isogyres presenting with an evident cross-shaped pattern, grossly aligned with the fixation axis, were observed in all patients with centers within the pupillary dark area, impeding the exact determination of the center point. During the rotational scan in 78.3% of the eyes the cross-shaped pattern of the isogyre gradually separated to form two distinct hyperbolic arcs in opposite quadrants, reaching their maximal separation at 45 degrees with respect to angle of cross-shaped pattern formation. The corneal cross and hyperbolic-pattern repeated every 90° throughout the 360° rotational scan. While the interpretation of the isogyres presents particular difficulties, two summary parameters can be extracted for each cornea: the presence/orientation of a single or two dark areas in post-processed images and isochromes. However, the development of dedicated software for semi-quantitative analysis of these parameters and enantiomorphism may become available in the near future. The possible application of polarimetric interferometry in the field of both corneal pathologies and corneal surgery may be of great interest for clinical purposes.

## Introduction

The healthy human cornea is a structure that is almost perfectly transparent to visible light, thus aiding image-forming light rays to pass to the retinal plane. The corneal stroma is the major component of the corneal structure accounting for its mechanical strength and optical properties.

It represents 90% of the corneal thickness and is formed by fibrous connective tissue containing collagen fibrils that have an average diameter of approximately 25–30 nm. The fibrils are super-organized in lamellae. Stromal transparency is guaranteed by a high degree of regularity of the structural organization of the collagen fibrils and, at a higher level, of the collagen lamellae. [1, 2] Several studies investigated the orientation of the collagen fibrils within the human corneal stroma using different methods such as X-ray diffraction and scanning and transmission electron microscopy [3–6], suggesting that the cornea is structurally anisotropic with a preferential orientation of collagen fibrils along the inferior-superior and temporal-nasal directions. The concept of a preferred orientation of the corneal fibrils was also supported by recent investigations that used techniques such as scanning laser polarimetry. [7–9] Based on corneal anisotropic characteristics it was proposed that the cornea behaves optically as a biaxial birefringent crystal. [10–13]

As with uniaxial and biaxial anisotropic crystals, when crossed polarizers systems are used to image the human cornea (i.e., polarized light is reflected from the cornea and detected through a second polarizer) interference pattern in the form of a dark cross-shaped (called isogyres) viewed against the iris, can be observed. [10, 14, 15] Variations in the directions of reflection of the incident plane, polarised rays and the amounts of rotation of their planes of polarisation influence the amount of light that will passes through the second polariser (analyser) modifying the resultant interference figure. [15] Thus, the corneal cross is the cumulative result of rotation and retardation of light due to the corneal layers of collagen. [14]

The isogyres are areas of the corneal stroma where polarization of the incident rays remains unchanged, while the lighter zones between the cross arms correspond to corneal stromal areas where the incident light is rotated or depolarized. It was reported that other types of chromatic corneal interference figures, visible as coloured rings (isochromatics), can be produced by detecting reflected polarized light through a second “crossed polarizer”. [15] These isochromatics vanish when at least one of the polarisers are removed from the optical path of the observation system.

In the present study we investigated, by using a computerized rotating polarimetric interferometry system based on a cross-polarized technology, the detection and the morphological characteristics and features of the interference figures produced by the intact corneal stromal structure in healthy human cornea.

## Materials and methods

**Fig 1:**
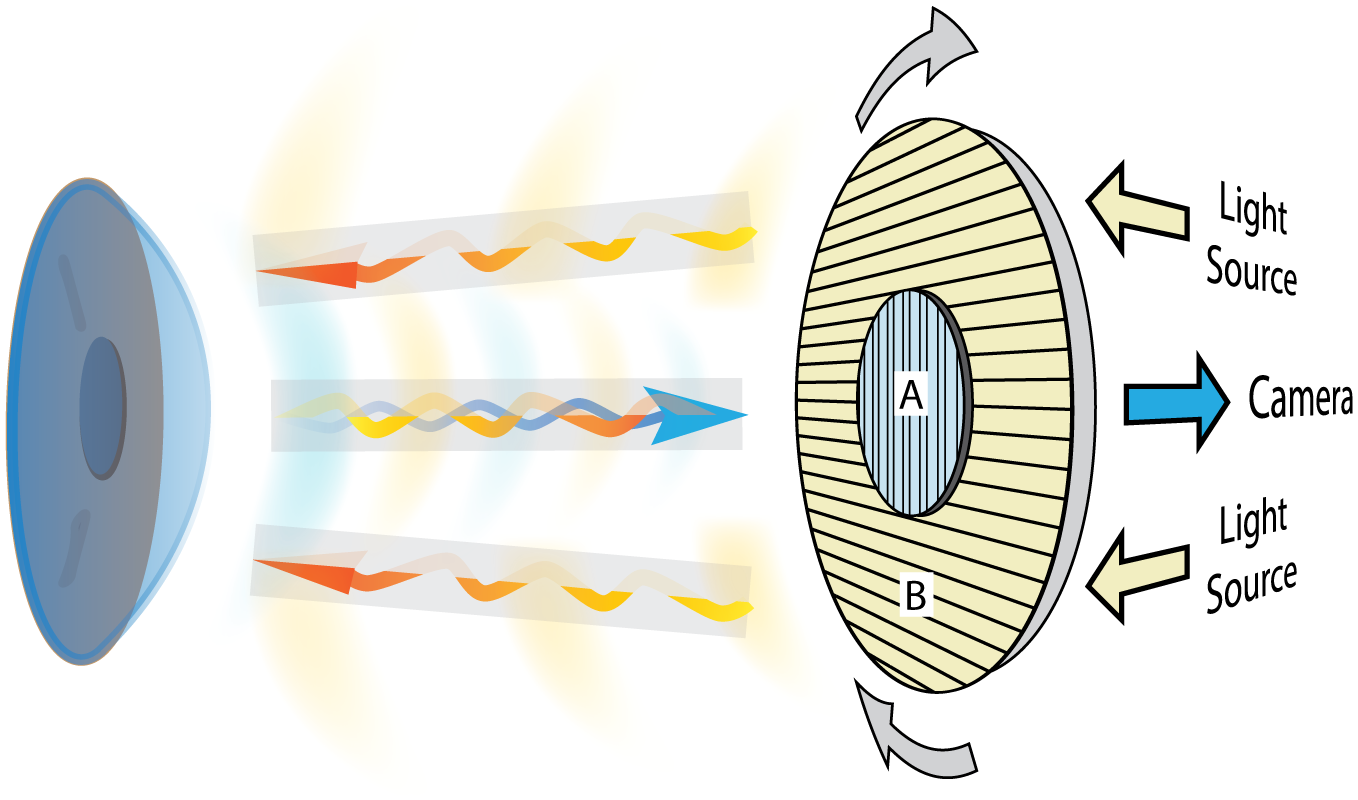
Schematic representation. Principle of polarimetric interferometry of the corneal stroma. The incident light projected by the LED light source passes through the first polarized filter (B) and illuminates the cornea. The backscattered light after interaction with corneal tissue passes through the second cross-polarized filter (A) and is captured by the detector camera.

A novel medical device specifically designed to evaluate the effects of in vivo interaction between polarized light and corneal stroma (LUMAXIS^®^—Phronema srl Lumaxis^®^, Italy) was used to evaluate healthy human corneas. This non-contact instrument was designed to record the interference phenomena observed when polarized light passes through a birefringent optical medium (such as the corneal stroma), positioned between two cross polarizers. This technique is called polarimetric interferometry. A schematic representation of the polarimetric interferometer used in this study is shown in Fig 1. The system used in our study employs a patented optical design made up of five concentrically arranged components: a ring of 30 white light emitting diodes (LED; cool white 6000K) (1), a rotating ring (2) to a linear polarization filter (3) is attached to the outer portion and a second linear polarization filter (4) set at 90° with respect to the first occupies the central portion. A high-resolution color CCD sensor (5) is positioned at the center of the LED ring and behind the second filter. The ring permits the rotation of the linked crossed polarizers (LCP). We modified the standard version by setting the light source/filters/camera unit on a stable base and by adding a compass in order to measure the angle of orientation of the linear polarization positioned in front of the light source.

Patient acquisitions comprised a sequence of images corresponding to different angles of the polarization plane that were obtained by the rotation of the LCP through 360°, producing a complete corneal scan. All raw images were elaborated with the integrated software. The acquired images were post-processed using a novel algorithm. Briefly, the linear stack alignment SIFT plugin of ImageJ [16] was used to align single images. A single image was then produced from all images based on the minimum background illumination which was then subtracted from each individual image. The sequence of images thus obtained showed the areas of the cornea where polarized light changes its polarization plane as the LCP was rotated during the scan. Black background represented the areas where light was unmodified from the original polarization angle. A binary threshold was then applied to yield a summary static images (SUM) in which the dark zones represent the areas where the polarization of the light did not change during the acquisition.

In order to analyze the polarimetric behavior of the cornea in the healthy population, normal subjects were enrolled in this observational study after complete ophthalmological examination. Inclusion criteria were: best corrected visual acuity ≥ 0.1 (LogMAR), normal biomicroscopic examination of the anterior segment, transparent cornea and crystalline lens, “prolate-shaped” cornea or regular symmetric topographic pattern of astigmatism (less than 0.75 D), with regular morphology of the posterior corneal surface. The exclusion criteria were: positive history for systemic or ocular pathologies, contact lens use, intra ocular pressure above normal limits, and corneal curvature outside normal limits (< 41 and > 45 D).

Each patient was examined in sitting position, with standardized (dim light) room illumination, without additional light sources. The circular 10 mm region of analysis provided by the instrument software (and visible on the systems monitor) was carefully aligned with the circumference of the corneoscleral limbus, in order to obtain a centered image of the cornea. Each cornea was scanned by rotating the LCP placed in the acquisition head obtaining the complete (360°) image sequence of all meridians, for a total examination time of less than 30 seconds. The software acquisition frame rate is 15 frames per second. A minimum of 80 images for each session were acquired and data were subjected to the aforementioned elaboration protocol with the ImageJ software. The morphology and the changes of the observed interference figures were analyzed in each patient for a complete rotational scan (0° to 360°).

Three sequences of images were acquired for each eye. Each sequence was processed with the described software protocol and the morphological results were assessed by two observers in consensus. A third observer was consulted in case of disagreement.

## Results

Twenty-three normal subjects (46 eyes) were included. The 11 females and 12 males had a mean age of 35.2±5.3 years (age range: 26–45 years). None of the subjects had ocular or systemic diseases nor used contact lens or any medications. The mean spherical refractive error was -0.34 ± 0.20 (range +0.50 to -0.75) and the mean cylinder error was -0.40 ± 0.12 (range 0 to -0.75). The mean central corneal thickness (CCT) was 538 ± 23 μm (range 508 to 574 μm). The mean corneal power (mean keratometric measurements, K_mean_) performed with the HR Pentacam (Oculus, Wetzlar, Germany) was 43.23 ± 0.65 (range 41.75–44.25).

In every patient, we evaluated the geometric features of the polarimetric interferometry corneal interference figures (isogyres), their symmetry and variations observed during the rotation of the LCP. Isochromes morphology was also evaluated regarding geometrical shape and colors appearance.

**Fig 2:**
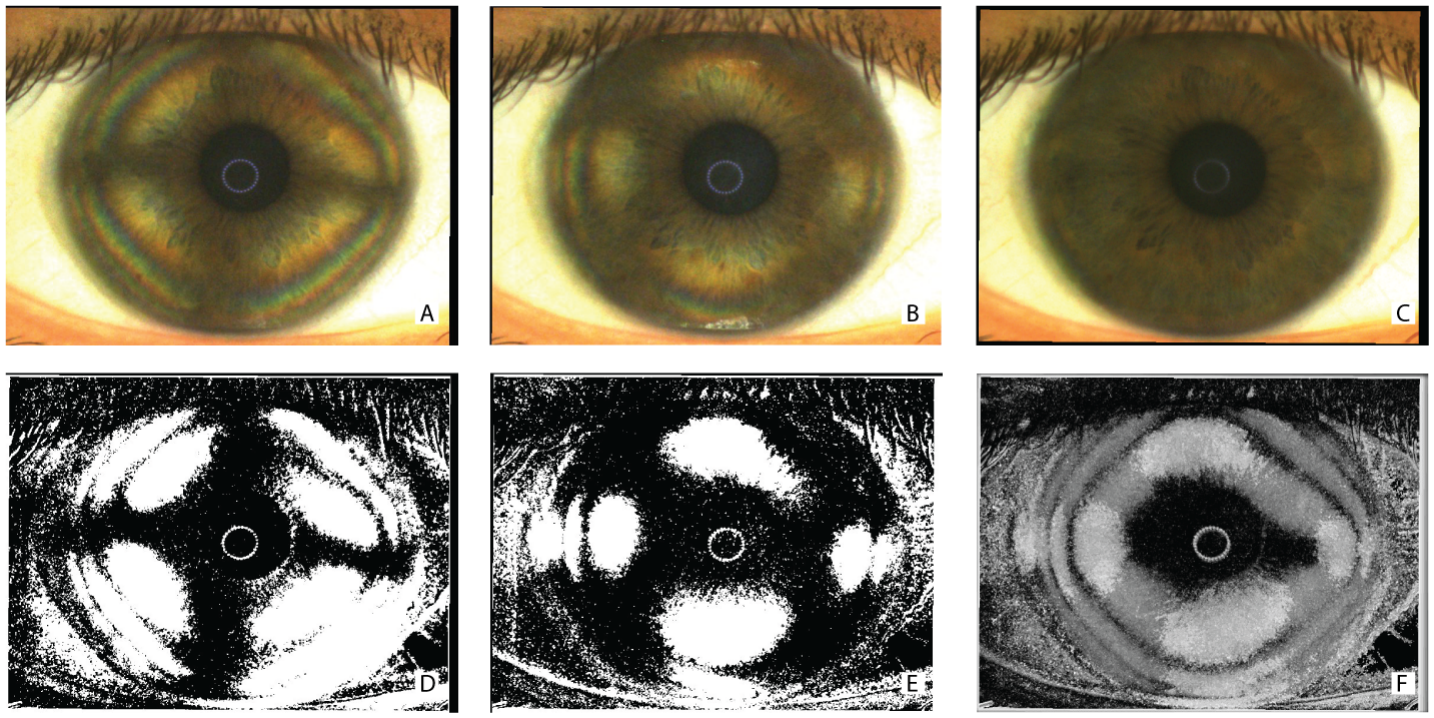
Polarimetric interferometry images of the cornea obtained during acquisition and after software elaboration. Frames of the acquired raw sequence of a single representative subject (A and B) showing the corneal cross-shaped pattern (A) and the hyperbolic-shaped pattern (B) of the interference figures. The entire sequence of at least 80 images was used to calculate a background illumination image (C). The aforementioned frames were reproduced after subtraction of background luminance and threshold filtering (D, E). A final summary static image (SUM image) was then calculated by further post-processing of the entire sequence and indicates areas where light does not change its polarization during the acquisition (F).

The characteristic corneal isogyres presenting with an evident cross-shaped pattern, grossly aligned with the fixation axis, were observed in all patients. Fig 2 shows the raw (Fig 2A–2C) and post-processed (Fig 2D–2F) images of a representative subject. Corneal isogyres appeared as dark figures produced by the lower level of light radiation detected by the sensor, with a background of brighter areas (between the cross-arms) in each image. The corneal cross centers were found to fall inside the pupillary dark area, without appreciable differences that could impede the exact determination of the center point.

The interpretation of the image characteristics by the first two observers was always in agreement. We did not observe detectable morphological differences, in the polarimetric interferometry images, with respect to the corneal power and refraction of the examined subjects.

**Fig 3:**
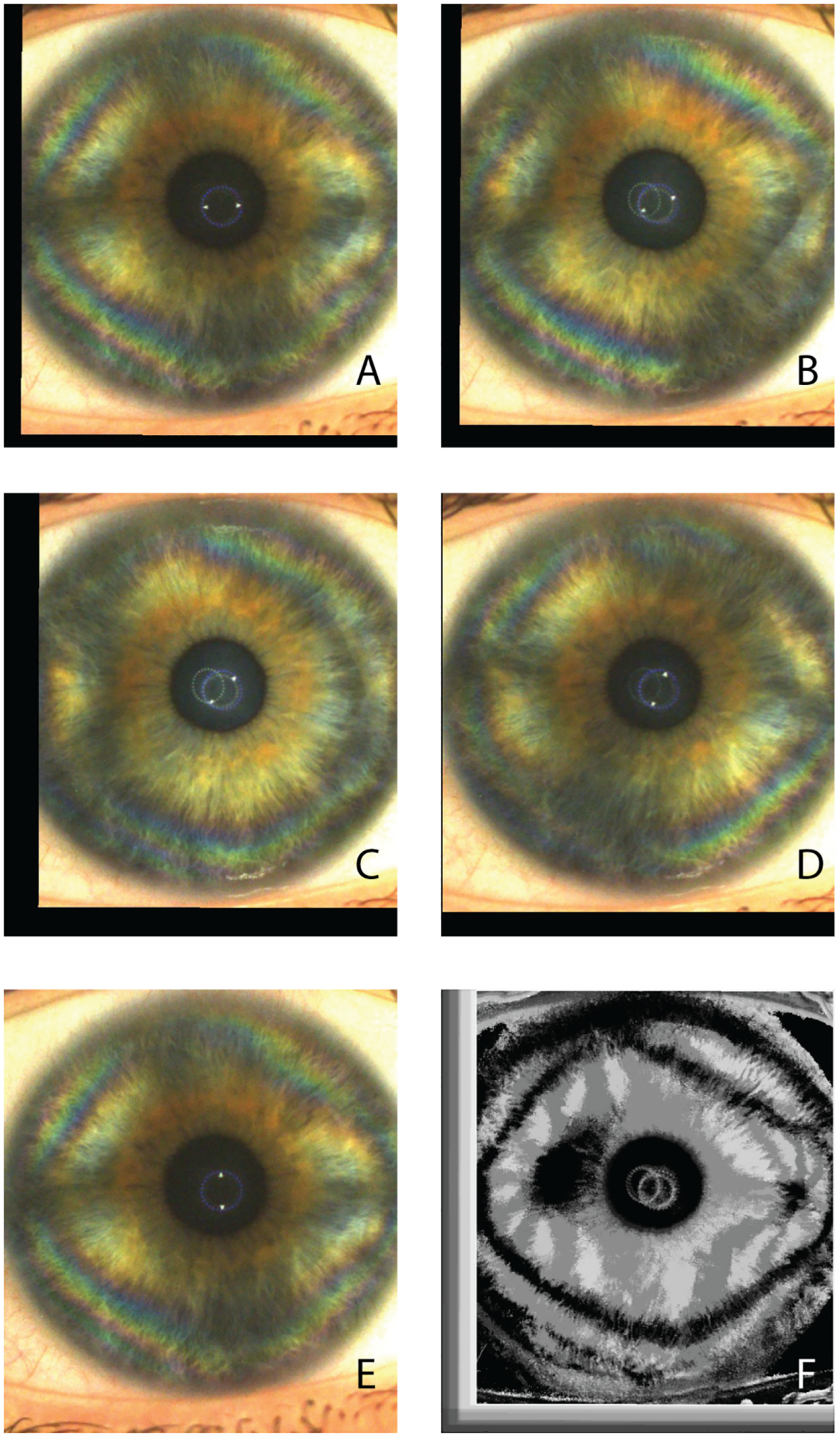
Corneal polarimetric interferometry of type B pattern. Frames of a representative raw sequence (A-E) in a case in which the isogyre gradually changes its corneal cross pattern (A) to form two distinct hyperbolic arcs in opposite quadrants (B-D). The maximal separation is at 45 degrees with respect to angle of cross-shaped pattern formation (C). At 90° rotation of the LCP with respect to frame A, the cross-shape pattern is again visible. (F) The final summary static image (SUM image) is characterized by a separation of the figure into two distinct zones localized in the corneal mid periphery along the horizontal meridians. Peripheral isochromes are visible on individual photograms.

**Fig 4:**
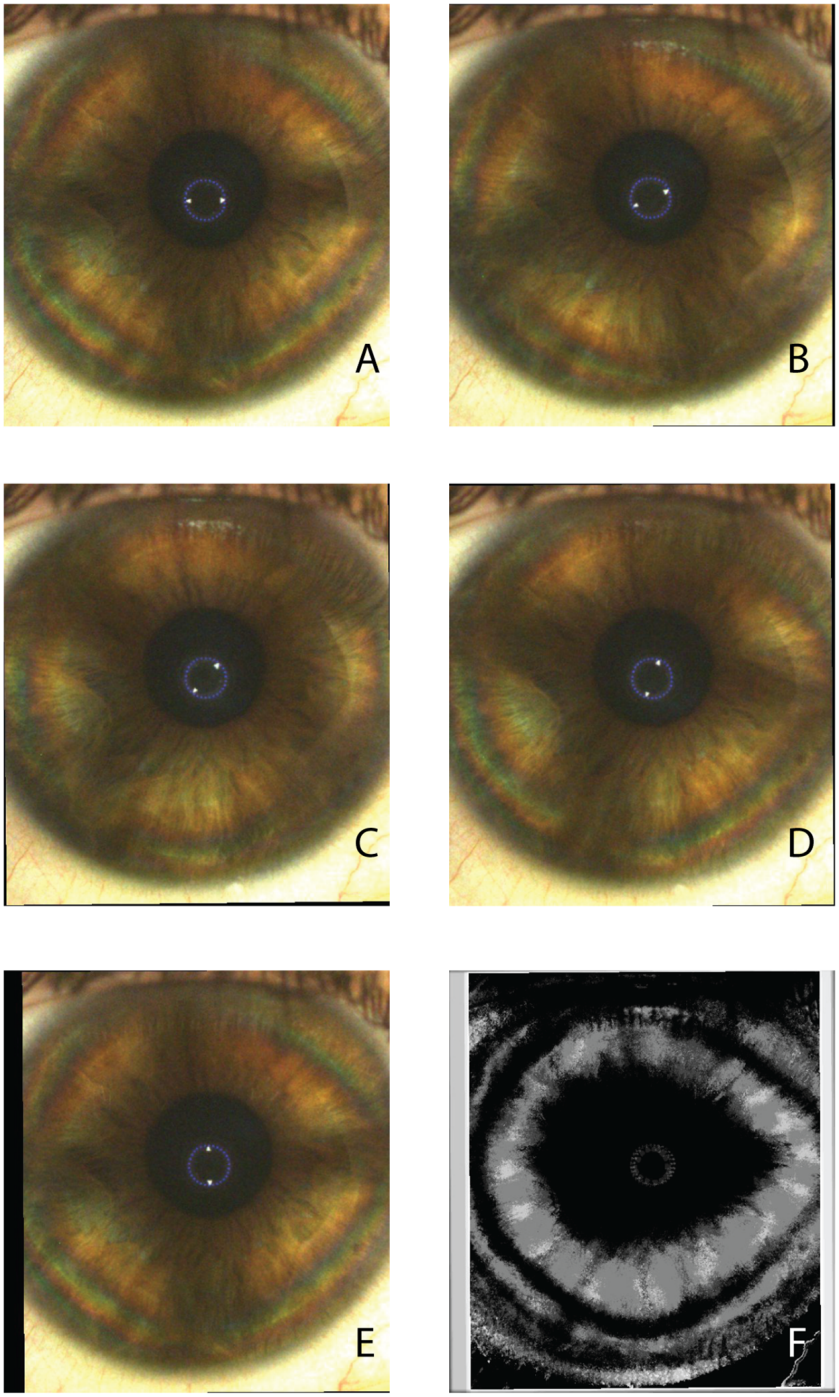
Corneal polarimetric interferometry of type A pattern. Frames of representative raw sequence (A-E) in a case in which the isogyre maintains its corneal cross-shaped pattern with the rotating scan of the LCP. (F) The final summary static image (SUM image) is characterized by a single “pear shaped” dark area with the major axis along the horizontal meridians. Peripheral isochromes are visible on individual photograms.

The formation of a corneal-cross image was observed in all subjects between 0° (horizontal meridian which is the starting position of the LCP during the examination) and -15° in the right eyes and between 0° and +15° in the left eyes, therefore indicating a slight inferior angular shift of the nasal cross horizontal arm. The isogyre interference pattern of the corneal cross was found to be periodic, repeating its morphology every 90° of rotation of the LCP. (Figs 3A–3E and 4A–4E) The observed image sequences between 0° to 90° were identical to the sequences recorded between 90°-180°, 180°-270° and 270°-360°.

During the LCP rotation, we observed morphological changes of the interference figures. In 78.3% of the eyes the cross-shaped pattern of the isogyre gradually separated to form two distinct hyperbolic arcs in opposite quadrants, reaching their maximal separation at 45 degrees with respect to angle of cross-shaped pattern formation (Fig 3A vs 3C). Again, the hyperbolic-pattern repeated every 90° throughout the rotational scan of the LCP.

**Fig 5:**
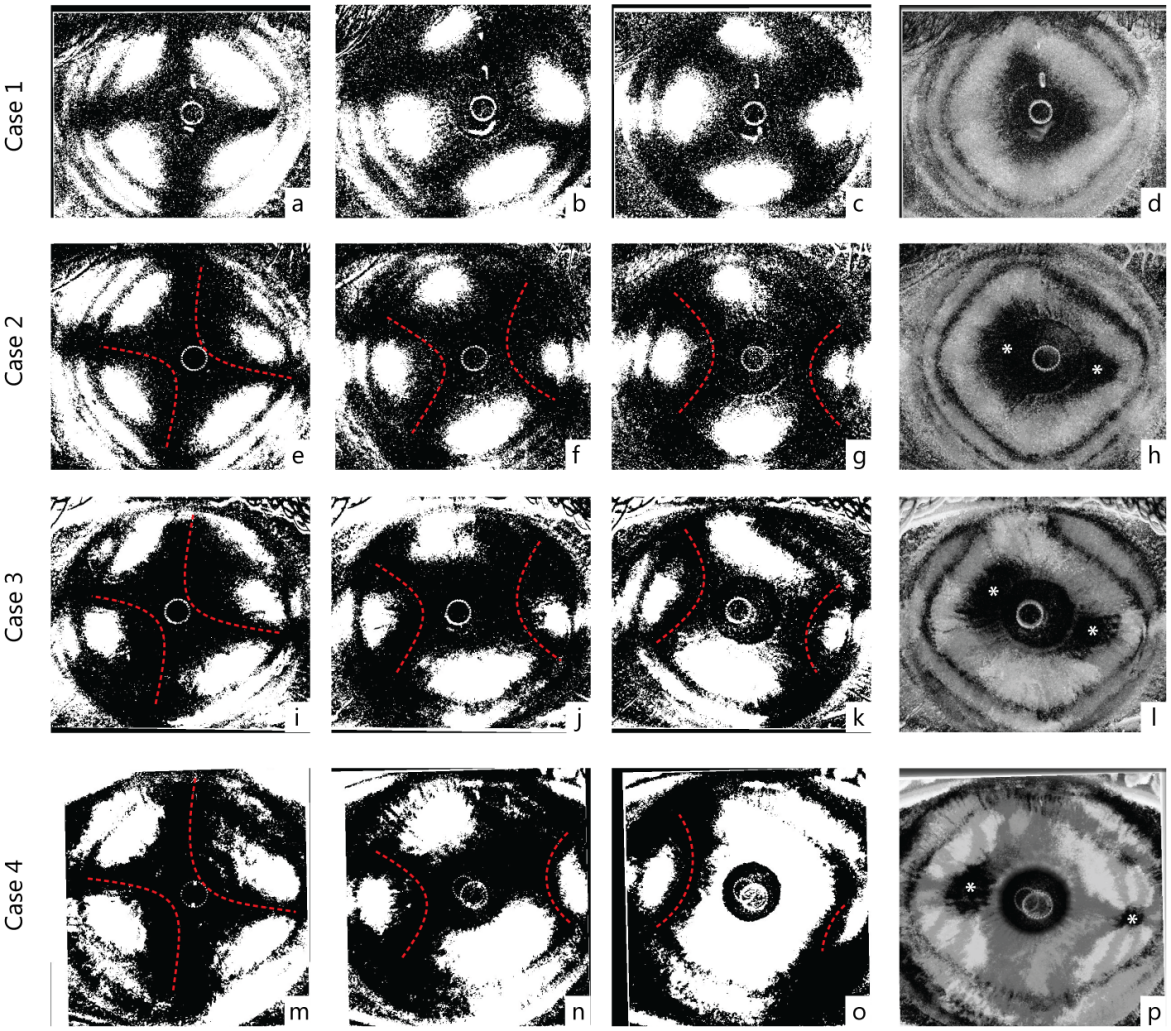
Processed images of four representative cases (right eyes). The first row (case 1: a-d) shows a type A pattern while the last row represents a typical B pattern (case 4: m-o). Intermediate rows (case 2: e-h, and case 3: e-l) show two patients with intermediate appearance of interference figures, classified as B pattern because of two distinct optic axis (foci) in the SUM image (h, l). Starting from the left, the first column represents the maximum cross-shape pattern appearance (e, i, m), the second an intermediate phase (f, j, m) at 22°30' degree of rotation and the third the maximum hyperbolic arcs separation (g, k, o) at 45°. The last column shows the SUM images (d, h, l, p). Dashed red lines highlight the profiles of the hyperbolic arcs in clearly biaxial cases (Type B pattern) (case 2, 3 and 4). Asterisks mark possible location of the interference figure optic axis (foci).

In 21.7% of the eyes the visible isogyre maintained its cross-shaped morphology without a distinct separation of the cross arms with the rotation of the LCP (Fig 4A–4E), although visible deformations of the cross-shape were present during the rotation of the scan, as shown in Fig 5.

Analysis of the SUM images where dark zones represent areas where light does not change its polarization during the acquisition revealed different morphological patterns. In 22% of the cases, the SUM images were characterized by a single grossly “pear shaped” dark zone (type A pattern), therefore exhibiting the characteristics of uniaxial samples (see Figs 2F and 4F). Conversely, in the cases where hyperbolic arcs formation occurred (78%), a clear tendency to produce a separation of the figure into two distinct zones was observed (Figs 3F, 5H, 5L and 5P). This pattern (type B pattern) indicates characteristics of bi-axial structures. The two distinct dark zones in the SUM images were located along the horizontal meridians of the cornea (maintaining the described angular orientation with nasal-inferior shift between 0° and 15°), the larger one temporally and the smaller one nasally, and tended to increase their separation towards the periphery in proportion with the degree of hyperbolic arc-shape separation of the isogyres.

**Fig 6:**
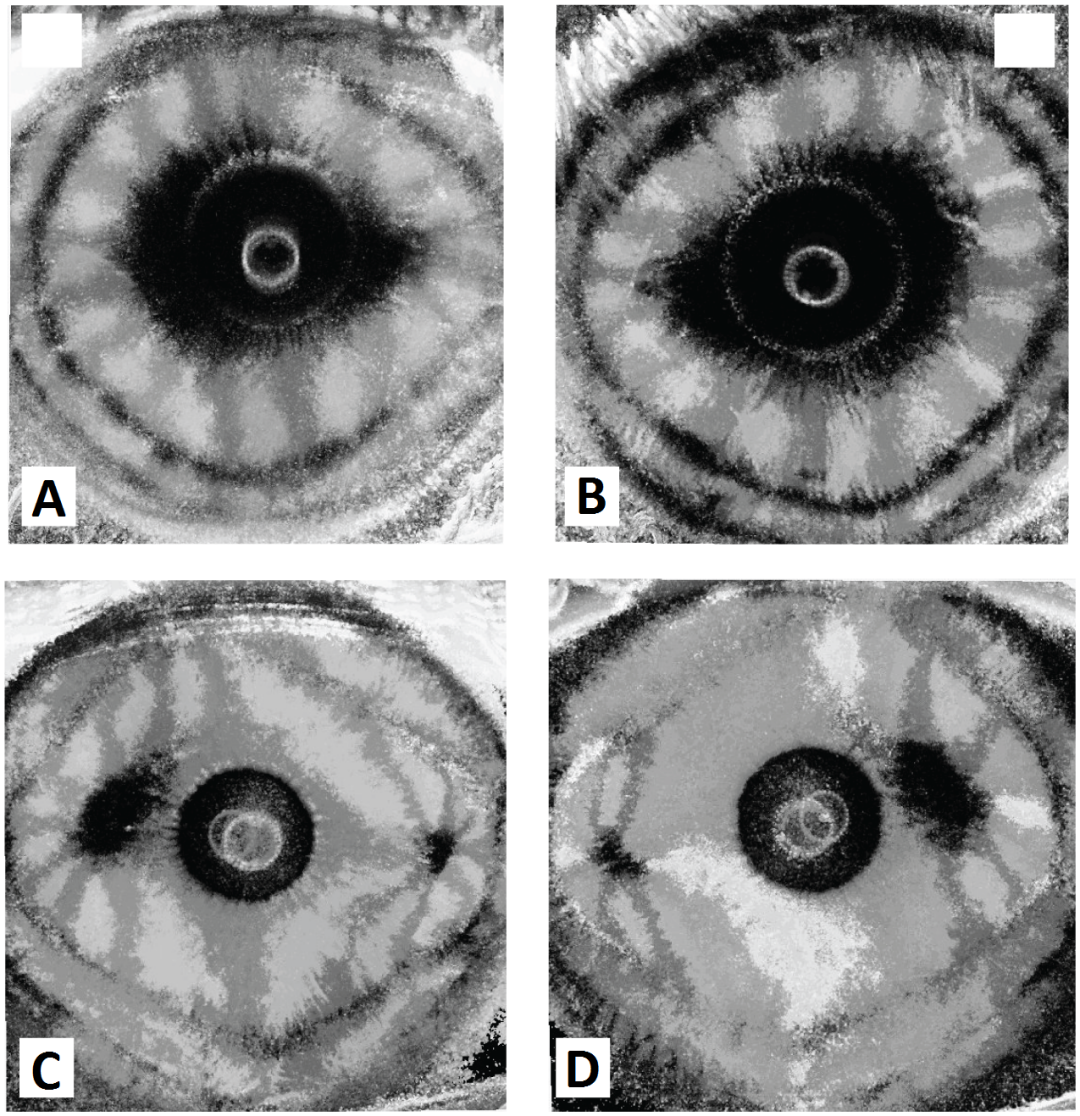
Enantiomorphism phenomenon in corneal polarimetric interferometry. Summary images that illustrate corneal interference pattern enantiomorphism of the right eye (RE) and left eye (LE) of two representative patients. (A, B) “Pear shaped” uniaxial pattern (type B). (C, D) Double asymmetric peripheral pattern (type A): note that the interference patterns present nasal-inferior shift between + 15°and—15° (respectively for the right and left eye) with respect to the horizontal meridian (0°–180°).

When comparing images for the right and left eye of each examined patient an evident pattern of enantiomorphism was observed: images were specular along the vertical axis, similar but not superimposable as presented in representative example shown in Fig 6. Right and left eyes of the same patient always showed identical type “a” or “b” pattern. In both uniaxial (Fig 6A and 6B) and biaxial pattern (Fig 6C and 6D), a nasal-inferior shift of the image horizontal axis was observed.

**Fig 7:**
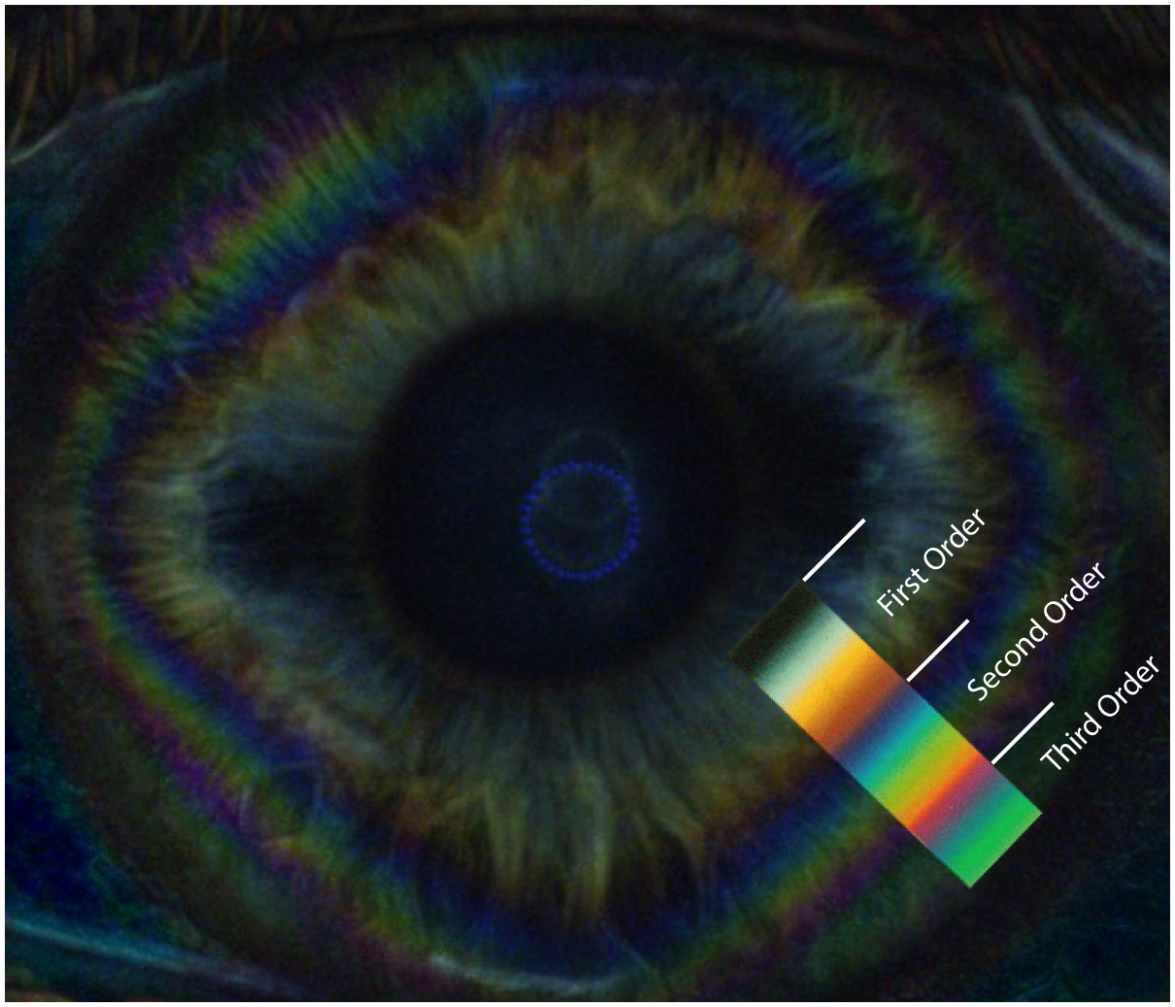
Corneal isochromatic interference figures in polarimetric interferometry enhanced after subtraction of background image. Colored image showing the peripheral corneal isochromes, with a rounded square morphology. The matching changes of colour bands of the 1^st^ through 3^rd^ order of the Michel-Levy colour sequence are shown in the colour overlay.

In all examined patients, other characteristic chromatic interference figures were observed in raw images of each eye (Fig 3A–3E and 3A–3E). Mid-peripheral and peripheral corneal bands, or diamond-shaped coloured fringes (isochromatic rings or isochromes) appeared as concentric circles generally distorted to form quadrilateral uniaxial figures grossly centered on the corneal vertex (Figs 3A and 4A). The sequence of color variations within these bands accurately corresponded to the Michel-Levy isochromes color chart [17] (Fig 7), up to the third order. No detectable differences in color ranges, shape or orientation of the isochromes were observed between patients. The features of the isochromes for each patient were not modified during the rotation of the LCP, except for cyclic fluctuations in intensity every 90°.

## Discussion

The corneal stroma represents the greater part of the corneal thickness, and is composed of collagen fibrils that are organized into regular lamellae. The peculiar lamellar structure formed by layers of parallel collagen fibrils combines the special requirement for the cornea to produce the strength necessary to withstand the tension in the globe of the eye counterpoising the intraocular pressure, while maintaining an almost perfect transparency. [18] The organization and distribution of these collagen lamellar structures accounts for important mechanical and optical properties of the human healthy and diseased cornea. [6, 19, 20] Different ex-vivo studies investigating the macrostructure and the collagen fibrils orientation of the corneal stroma have been conducted by using electron microscopy, X-ray scatter and second-harmonic-generation microscopy without the need to section or stain the tissue. [2–6, 21, 22]

Previous in vivo investigations described case reports in which the stromal structure was imaged analyzing the cornea between crossed linear polarizers, producing the characteristics dark cross (isogyres) against the background of the iris. [15] The origin of the corneal cross was attributed to the cumulative results of rotation and retardation of light due to the collagenous corneal layers, and therefore is dependent on the structure of the stromal fibrils and lamellae. [14,15] Interestingly, the isogyre interference figure is not observed when circularly polarized light is used. A recent study on human subjects used circular polarization biomicroscopy for determining the corneal stromal lamellar organization, in vivo, and reported that an approximate elliptic/hyperbolic distribution of stromal fibrils, interpreted as collagen, can be identified within central and paracentral zones of the cornea. [23]

In the present study, we used a computerized rotating polarimetric interferometer based on a cross-polarized technology, specifically designed for the imaging of the human cornea, in normal subjects. When applied to in vivo biomicroscopy of human corneas, this technique produced consistent findings in all subjects investigated. In all examined corneas, the in vivo polarimetric interferometry examination produced the characteristic interference figures termed as isogyres, seen as dark cross-arms produced by the lower level of light radiation detected by the sensor as a cumulative result of rotation and retardation of light as it passes through the layers of the cornea. [15] While the dark corneal cross has long been considered to be originated by birefringence, more recently it was suggested that the isogyre pattern in the eye lens and in the cornea depends predominantly on refractive effects while polarized light passes through the tissues. [15, 24, 25]

In our study, the shape of the corneal cross appeared to be similar in all subjects and was characterized by a “maltese cross” [25] morphology grossly centred on the corneal vertex, although the dark background of the pupillary area impeded the exact determination of the cross centre. Typically, with the rotation of the LCP the isogyre morphology changed periodically (repeating its morphology every 90° of scan rotation) according to two main patterns. In 22% of the cases the isogyres maintained, overall, the shape of a single dark corneal cross along the 360° rotation of the scan, while in the majority of eyes (78%) the corneal cross gradually separated to form two distinct peripheral corneal hyperbolic arc in opposite quadrants, during the rotation of the scan. These arcs reached their maximal separation at 45° with respect to the angle of the corneal cross formation and these patterns again repeated every 90° of scan rotation. These rotational variations of the isogyre morphology were evaluated by using a post-processing pipe-line that applies a binary threshold to the entire sequence of images and produces a summary static image. The cross-arms and the hyperbolic-arc shape figures represent the areas where the polarization plane of the light does not change during rotation of the polarized filters. The pipeline generated summary static image provides a graphical representation of the areas where the polarized light is unaffected during the examination, and the the dark zones indicate the corneal areas where reflected light was unmodified. This type of interference figure generation is similar to the polarimetric interferometry imaging of crystals in optical mineralogy where uniaxial minerals show a cross-shaped figure centred on the optical axis while biaxial minerals produce hyperbolic arc figures opposite to each other, that varies with the rotation of the scan. [11–13]

In the cases in which the corneal cross-shape of the isogyre was maintained during the scan, the summary static images produced a single dark central “pear shaped” pattern (type A pattern), therefore presenting the interference pattern typical of uniaxial structures. We hypothesized that this pattern indicates that the collagen lamellae orientation produces a single centred axis of polarized reflected light by the stroma itself. Confirmation of this hypothesis will require further studies. In the majority of cases in which the corneal-cross separated to form two distinct peripheral hyperbolic arcs the summary static images produced a double dark spot (supero-temporal and infero-nasal) located approximately mid-way between the corneal centre and the limbus (type B pattern). This type of figure represents the interference pattern of biaxial structures (two foci), with elliptical/hyperbolic fibrillar distribution in the stroma. [23]

The distance, size and position of the two dark zones, (foci) observed in the type B pattern was found to be dependent on the degree of separation of the corneal cross into two hyperbolic arcs observed in the sequence of images acquired during the rotating polarimetric interferometry scan. As shown in Fig 5, the corneal cross separated into hyperbolic arcs in the majority of cases, but different degrees of separation were observed: from no separation characterized by a pear-shaped single dark image in type A pattern, to maximal separation producing two distinct peripheral dark foci in type B pattern. Intermediate values of separation were characterized by position of the two dark zones closer to the central corneal area. However, in consideration of the interference of the dark pupillary area that precludes the visualization of fine details of the dark zones in the central cornea, in our images, it was not possible to determine whether in type A pattern, a minimal degree of separation in the centre was present. Therefore, we cannot exclude the possibility that all interference figures should be interpreted as various degrees of type B pattern (images of biaxial structures) and that the pear-shaped appearance (type A) could be related to the proximity of the two optical axes. The degree of separation of the two axes could become a novel parameter to investigate in corneal pathologies, since it is probably determined by the internal stromal lamellar organization intrinsic of each cornea.

The observed figures show a high degree of similarity to the ones described by Mission [23] who described, using circular polarization biomicroscopy in vivo under high magnification, two distinct dark patches representing the foci of the elliptic/hyperbolic distribution of corneal stromal fibrils. Another similarity with the previous investigation, [23] is that the two dark spots observed in our study analyzing the summary images, showed the tendency to be aligned along nasal/inferonasal to temporal/superotemporal axis at approximately 15° from horizontal. This consistent finding observed in all subjects may indicate a similarity in the orientation and organization of the corneal stromal lamellar structure, as observed by polarimetric interferometry, to the results previously reported using circular polarization biomicroscopy. [23]

Interestingly, pairs of eyes showed approximate mirror symmetry and this feature can be interpreted as a clear characteristic of physiological enantiomorphism in the healthy human cornea, being images of right and left eyes specular along the vertical axis, but not perfectly superimposable. Although the corneal shape is known to vary significantly from individual to individual, the presence of topographical enantiomorphism exhibited by fellow corneas in humans has been already reported by using different imaging techniques such as corneal topography, wavefront aberration and x-ray scattering corneal analysis. [26–29] The characteristics of corneal enantiomorphism observed by the polarimetric interferometry analysis, in our study, well matches with the established knowledge that the surface topography of fellow normal human corneas exhibits a degree of non-superimposable midline symmetry. [29] The structural origin of this characteristic is not fully understood, but it is believed that the precise organization of stromal collagen plays a role in determining corneal shape and normal range of asymmetry between right and left eyes, necessary for optimal optical properties in human eyes.

Finally, corneal polarimetric interferometry in our study indicated that every examined patient showed the presence of “isochromes”, presenting as peripheral coloured rings. [12, 15, 23] The origin of this chromatic interference figures, which vanish when one or both polarisers are removed, [15] is known to be dependent on corneal birefringence. These coloured, approximately diamond-shaped, rings with horizontal and vertical apex are formed due to a graded rise in corneal peripheral birefringence, that causes increasing retardation of incident polarized light and corresponded to the ones reported by previous studies that used circular polarization biomicroscopy [23]. In our observations, the isochromes were located in the corneal paracentral and peripheral zones, while the centrifugal progression of color sequence within the corneal isochromes matches that expected up to third-order interference of the Michel-Levy isochromes color chart. [17] The presence of corneal isochromatics, with the described characteristics, with polarimetric interferometry analysis can be considered as a normal feature in the healthy human cornea, that expresses the physiological gradient of corneal birefringence.

To our knowledge, this is the first systematic description of all possible parameters in interference patterns originating in corneal stromal in-vivo analysis with polarimetric interferometry in the normal human cornea. It would be of interest to ascertain whether this in vivo analysis may be useful as a diagnostic tool in the clinical assessment of corneal pathologies.

A recently published case series evaluated the application of polarimetric interferometry in normal and diseased corneas. [30] The authors reported that that the cross-like image of the cornea was indeed detectable, despite the quality of the perceived images was affected by stromal transparency and curvature changes in different pathologic conditions, including keratoconus, corneal oedema and stromal scars. However, the study lacked a detailed systematic description of the interference figures, reported in the present study. We observed specific morphological characteristics in condition of normality, justifying further studies concerning its diagnostic value in clinical conditions that affect the corneal structure resulting in alterations of the polarimetric interference patterns.

In fact, the origin of these figures is known to be dependent on the peculiar organization of the collagen lamellar structure and organization of the corneal stroma and based on our preliminary observations the morphological characteristics of the corneal isogyres (corneal cross), and their rotational variations, can be easily observed in healthy transparent corneas in vivo, by using this technology. The main limitation of the current technology is represented by the absence of quantitative/numerical data, being the interpretation of the examination solely based on morphological examination of the interference figures. While the interpretation of the isogyres presents particular difficulties, two summary parameters can be extracted for each cornea: the presence/orientation of a single or two dark areas in SUM images and isochromes. However, the development of dedicated software for semi-quantitative analysis of these parameters and enantiomorphism may become available in the near future. The possible application of polarimetric interferometry in the field of both corneal pathologies and corneal surgery may be of great interest for clinical purposes.

Diseases affecting intra-lamellar and inter-lamellar organization (e.g. keratoconus) can influence the transmission of polarized light through the corneal stroma. The ability to distinguish normal from diseased cornea and the influence of other variables (e.g. age, gender, corneal thickness, refraction etc.) in a wider range in the normal population should be the objective of further investigation.
